# Inhibition of checkpoint kinase 1 potentiates anticancer activity of gemcitabine in bladder cancer cells

**DOI:** 10.1038/s41598-021-89684-5

**Published:** 2021-05-13

**Authors:** Makoto Isono, Kazuki Okubo, Takako Asano, Akinori Sato

**Affiliations:** Department of Urology, National Defense Medical College, 3-2 Namiki, Tokorozawa, Saitama 359-8513 Japan

**Keywords:** Cancer, Cell biology

## Abstract

Checkpoint kinases (CHKs) are involved in the DNA damage response in many cancer cells. CHK inhibitors have been used in clinical trials in combination with chemotherapeutics; however, their effect against bladder cancer remains unclear. Here, we investigated the efficacy of combining gemcitabine with MK-8776, a novel CHK1 inhibitor, in four bladder cancer cell lines. The effects of gemcitabine and MK-8776 on cell viability, clonogenicity, cell cycle, and apoptosis were examined alongside in vivo efficacy using murine xenograft tumor models. Combined treatment inhibited the viability and colony formation of bladder cancer cells compared to either single treatment. Although gemcitabine (10 nM) alone increased the cell number in S-phase, it increased the cell number in sub-G1 phase when combined with MK-8776 (0.5 µM). Combined treatment enhanced cleaved poly[ADP-ribose]-polymerase expression alongside the number of annexin-V-positive cells, indicating the induction of apoptosis. In vivo, administration of gemcitabine and MK-8776 was well tolerated and suppressed tumor growth. Mechanistically, the combined treatment elevated γH2A.X and suppressed Rad51 expression. Our study demonstrates that MK-8776 and gemcitabine combined induces apoptosis and suppresses proliferation in bladder cancer cells by inhibiting CHKs and DNA repair. Therefore, CHK1 inhibition combined with gemcitabine may be a potential treatment for bladder cancer.

## Introduction

Bladder carcinoma accounts for 7% of all cancer cases^1^, and approximately 30% of all newly diagnosed bladder cancers invade muscle, while 50% can quickly progress and metastasize^2^. Unfortunately, the clinical outcomes of systemic therapy in patients with locally advanced or metastatic bladder cancer are poor. Systemic cisplatin-based combination chemotherapies are the standard first-line treatments for bladder cancer^2^; however, despite an initial response rate of around 50%, many patients suffer tumor relapse within five years^3^. Furthermore, over half of all patients with bladder cancer display renal insufficiency, comorbidities, or frailty, and thus are ineligible for cisplatin-based chemotherapies^4^. The treatment options available for metastatic bladder cancer have recently improved due to the clinical development of second-line immunotherapies such as atezolizumab; however, patients with metastatic bladder cancer display partial or complete response rates of just 20–30% for checkpoint immunotherapies, and the median overall survival following atezolizumab treatment is approximately 7.9 months^5^. Therefore, further studies are required to identify a novel treatment strategy for patients with metastatic bladder cancer.

Since cells are exposed to stress by genotoxic agents, DNA repair pathways are required to preserve the genomic code^6^. Following DNA damage, checkpoint kinases (CHKs) arrest the cell cycle, providing time for DNA repair before cells with DNA damage enter mitosis. In response, transiently-activated ataxia telangiectasia mutated (ATM) binds immediately to DNA double-strand breaks (DSBs)^7^, while the presence of single-stranded DNA at stalled replication forks activates ataxia telangiectasia and Rad3-related (ATR), followed by CHK1^8^. Activated CHK1 phosphorylates various downstream effectors that cause the cell cycle to stall at the intra-S phase and G2/M phase until DNA damage is repaired^9^. To survive following DNA damage, p53 mutant cells rely on S or G2 checkpoints, in which CHKs have vital functions^10,11^, whereas cells with wild-type p53 can arrest the cell cycle at the G1 checkpoint for DNA repair. Notably, alterations in the p53 gene have been reported in almost all invasive bladder cancer cells^12^, and CHK1 protein expression has been associated with clinical–pathological characteristics in bladder cancer^13^. Therefore, CHK1 inhibition may abrogate cell cycle arrest and allow bladder cancer cells to enter mitosis with unrepaired DNA damage, leading to cell death.

Gemcitabine is a nucleoside analog that has been used as a chemotherapeutic agent for over 20 years. In cells, gemcitabine is phosphorylated and incorporated into replicating DNA strands, thereby terminating DNA synthesis, inducing DNA damage, and activating the DNA damage response^14,15^. Experimental CHK inhibitors have undergone early-phase clinical trials both as monotherapies and in combination with drugs that directly damage DNA. Consequently, the selective CHK1 inhibitor MK-8776 has entered clinical trials for the treatment of certain solid tumors and acute leukemia^16,17^; however, it remains unclear how CHK1 inhibition by MK-8776 triggers chemosensitization in bladder cancer cells. In this study, we investigated whether the co-administration of MK-8776 influences the cytotoxic effects of gemcitabine in bladder cancer cells and examined the possible underlying mechanisms.

## Results

### Inhibition of bladder cancer cell growth

**Figure 1:**
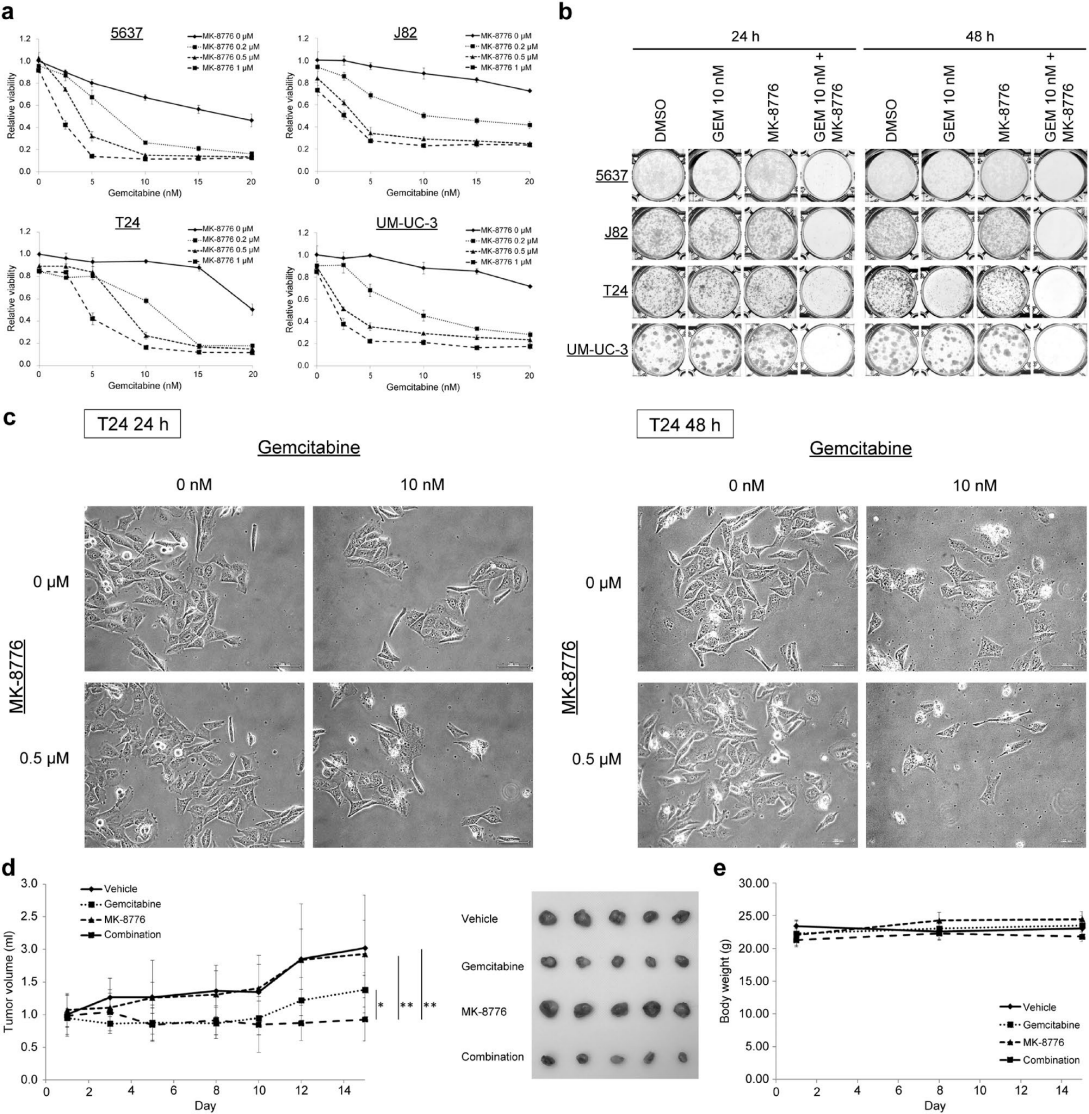
Viability and clonogenicity of bladder cancer cells after single and combined treatment. (**a**) Cell viability in 5637, J82, T24, and UM-UM-3 cells was determined by MTS assays (mean ± SD, *n* = 4) after 48 h. (**b**) Clonogenicity assays in bladder cancer cells treated with gemcitabine, MK-8776 (0.5 μM), or both compounds for 24 or 48 h. GEM, gemcitabine. (**c**) Morphology of T24 cells after treatment with gemcitabine and MK-8776. Scale bar = 100 μm. (**d**) In vivo efficacy of the combination treatment. UM-UC-3 cells were used for the subcutaneous tumor models. The vehicle group received DMSO and the other three groups received either gemcitabine (20 mg/kg), MK-8776 (4 mg/kg), or both (mean ± SD, *n* = 5, *p = 0.0593, **p = 0.0119). (**e**) Changes in body weight (mean ± SD, *n* = 5). No significant change in body weight was observed among the four groups at day 15.

We initially examined whether MK-8776 enhances the cytotoxic effects of gemcitabine in bladder cancer cells. The 3-(4,5-dimethylthiazol-2-yl)-5-(3-carboxymethoxyphenyl)-2-(4-sulfophenyl)-2H-tetrazolium (MTS) assay revealed that relative cell viability was lower in the four investigated bladder cancer cell lines treated with the combination of gemcitabine and MK-8776 than in those treated with each agent alone (Fig. 1a). In addition, the combination index (CI) value demonstrated that gemcitabine and MK-8776 exerted a synergistic effect (CI < 1) against bladder cancer cells under most treatment conditions (Table 1).

**Table 1:** Combination indices.

Gemcitabine (nM)	MK-8776 (μM)		
	0.2	0.5	1.0
**5637**			
10	0.214	0.113	0.085
20	0.246	0.199	0.185
**J82**			
10	0.436	0.322	0.335
20	0.659	0.488	0.543
**T24**			
10	0.419	0.730	0.836
20	0.821	0.851	0.884
**UM-UC-3**			
10	0.188	0.118	0.088
20	0.228	0.193	0.152

Cell viability was used to calculate combination indices (CI) for each investigated cell line using the Chou–Talalay method to determine synergistic effects (CI < 1 indicates synergy).

Similarly, combined treatment with gemcitabine and MK-8776 suppressed the colony formation of cancerous bladder cells (Fig. 1b, Fig. S1). Photomicrographs revealed marked morphological changes in bladder cancer cells treated with gemcitabine and MK-8776. After 24 h of treatment with gemcitabine alone or combined with MK-8776, bladder cancer cells were characterized by increased cell size and cell flattening compared to those treated with DMSO or MK-8776 alone, while the number of detached or shrunken cells increased after 48 h of treatment with gemcitabine and MK-8776, indicative of apoptosis (Fig. 1c, Fig. S2).

Next, we carried out in vivo experiments to evaluate the antitumor effects of treatment with gemcitabine and MK-8776 for 15 days. Combined treatment reduced the volume of tumor nodules relative to DMSO or MK-8776 alone; however, no significant difference in tumor size was observed between the gemcitabine and combination groups (Fig. 1d). Furthermore, mice treated with the combination did not display any remarkable weight loss (Fig. 1e). Thus, the combination of gemcitabine and MK-8776 was shown to inhibit bladder cancer cell growth both in vitro and in vivo.

### Gemcitabine and MK-8776 induce cell cycle disturbances

**Figure 2:**
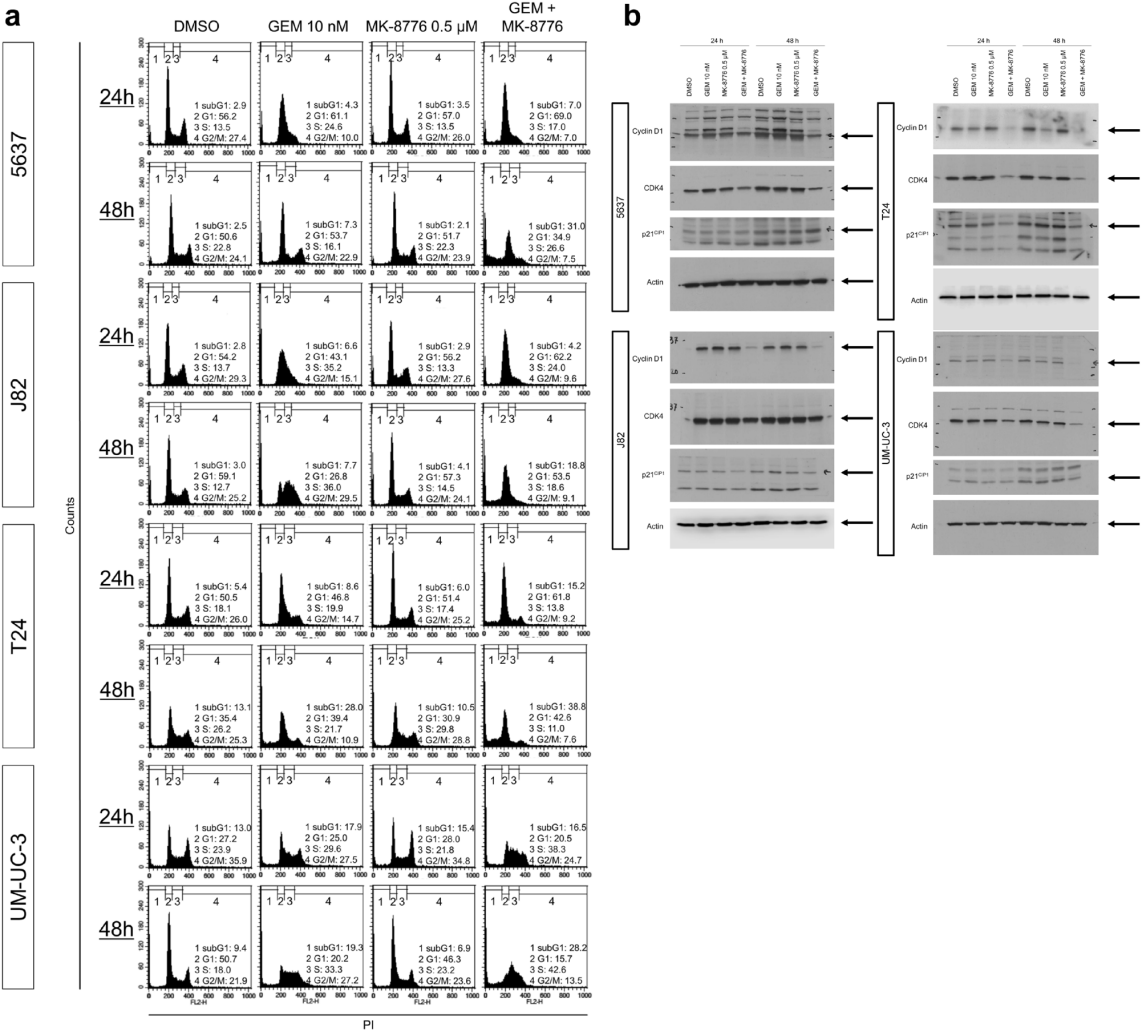
Changes in cell cycle distribution following treatment. (**a**) Flow cytometry analysis of the cell cycle following treatment with gemcitabine (10 nM) and/or MK-8776 (0.5 μM) for 24 or 48 h. DMSO was used as a solvent control. The relative distribution of the fractions is displayed. (**b**) Protein expression following the indicated treatment was evaluated by western blotting. Actin was used as a loading control. A quantification of the signals is illustrated in Fig. S3.

Next, we performed flow cytometry experiments to investigate the effects of the drug combination on cell cycle distribution. Gemcitabine alone and the combination treatment for 24 h increased the number of bladder cancer cells in S-phase (Fig. 2a, Fig. S3); however, this effect appeared to subside after 48 h of gemcitabine treatment alone. Conversely, the combination led to an increase in the sub-G1 fraction of all investigated cell lines after 48 h, whereas the sub-G1 fraction only increased slightly in the untreated controls or cells treated with gemcitabine or MK-8776 alone.

Consistently, western blot analysis revealed that gemcitabine and MK-8776 treatment for 48 h suppressed the expression of CDK4 and cyclin D1 in bladder cancer cells (Fig. 2b, Fig. S4). However, the combination treatment increased the expression of p21^CIP1^, a CDK inhibitor, in the 5637 cell line but decreased its expression in the other cell lines investigated. Together, these observations indicate that the combination treatment disturbs the cell cycle distribution and elicits apoptosis.

### Gemcitabine and MK-8776 induce apoptosis and necrosis in bladder cancer cells

**Figure 3:**
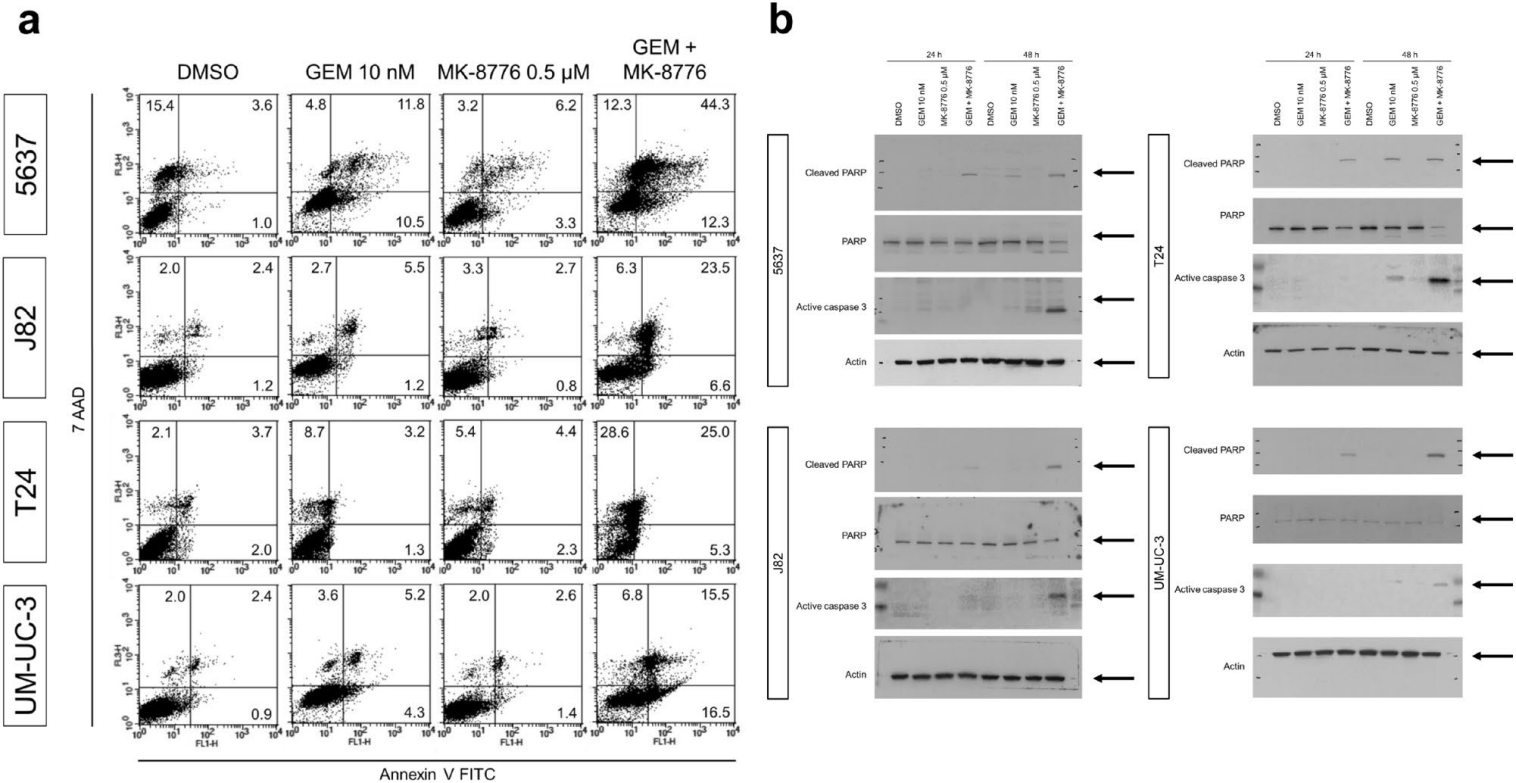
Cell death mechanism induced by the treatments. (**a**) Flow cytometric analysis of bladder cancer cells treated with indicated conditions after combined staining with Annexin V and 7-AAD. The results are expressed as a percentage of early apoptotic cells (lower right quadrant), late apoptotic cells (upper right quadrant) and necrotic cells (upper left quadrant). (**b)** PARP cleavage 48 h after the indicated treatments were assessed by western blotting. Actin was used as a loading control. A quantification of the signals is illustrated in Fig. S5.

To characterize the cellular effects of the drug combination in more detail, we investigated the induction of apoptosis. Annexin V/7-amino-actinomycin D (7-AAD) staining revealed that the combination of gemcitabine and MK-8776 increased the number of apoptotic bladder cancer cells (Fig. 3a, Fig. S5), while western blotting demonstrated that the expression of cleaved poly[ADP-ribose]-polymerase (PARP) was enhanced compared to either drug alone (Fig. 3b, Fig. S6). Therefore, these results indicate that the combination treatment elicits apoptosis in bladder cancer cells.

### MK-8776 enhances DNA damage induced by gemcitabine

**Figure 4:**
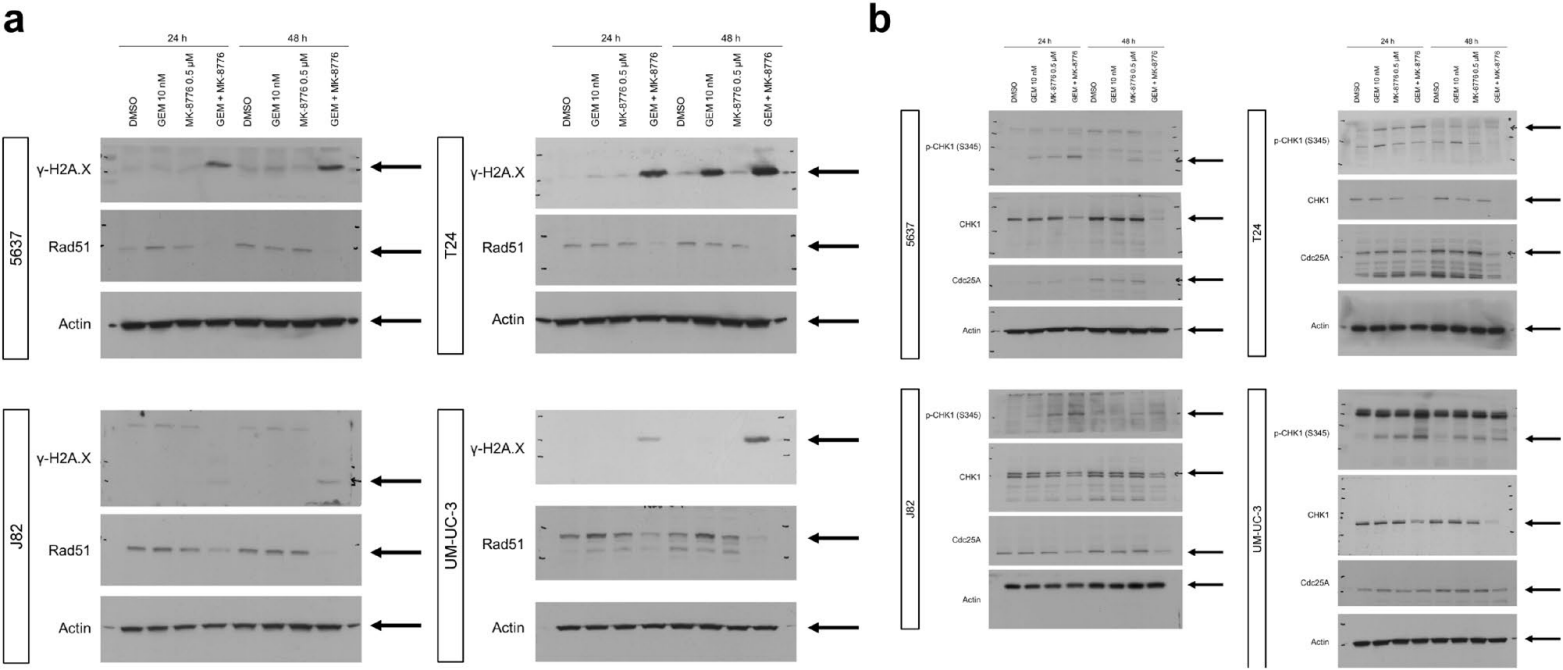
Western blot analysis of checkpoint factors. (**a**) γH2A.X and Rad51 protein expression following the indicated treatment. Actin was used as a loading control. A quantification of the signals is illustrated in Fig. S6. (**b**) Expression of cell checkpoint-related proteins and their phosphorylation in whole-cell lysates. Actin was used as a loading control. A quantification of the signals is illustrated in Fig. S7.

To determine the extent of DNA damage caused by gemcitabine and MK-8776, we analyzed whether the combination treatment induced the expression of γH2A.X, a DSB marker^18^, in bladder cancer cells by western blotting (Fig. 4a, Fig. S7). Exposing bladder cancer cells to the combination of gemcitabine and MK-8776 increased the expression of γH2A.X after 24 and 48 h. We also analyzed the expression of Rad51, which indicates homologous recombination repair activity. The combination treatment decreased Rad51 expression in bladder cancer cells, suggesting that exposure to the combination treatment decreased homologous recombination activity and the accumulation of DNA damage.

To confirm that MK-8776 inhibits CHK1 in bladder cancer cells, we investigated CHK1 signaling (Fig. 4b, Fig. S8). CHK1 phosphorylation at Ser345 was elevated after 24 h of gemcitabine-only treatment and further enhanced by cotreatment with MK-8776, indicating that the coadministration of MK-8776 and gemcitabine induces genotoxic stress. After 48 h, MK-8776 inhibited gemcitabine-induced CHK1 phosphorylation and decreased the expression of the downstream transcriptional target cdc25A, which dephosphorylates cyclin-dependent kinases and regulates the cell cycle. These results indicate that MK-8776 inhibits the activation of checkpoint signaling induced by gemcitabine treatment.

## Discussion

This preclinical study evaluated the suitability of the selective CHK1 inhibitor MK-8776 in combination with gemcitabine as a novel therapeutic approach for bladder cancer. Gemcitabine is a ribonucleotide reductase inhibitor that is incorporated into DNA and causes cell cycle arrest in S-phase^19^. Cell cycle checkpoints are activated by gemcitabine-induced DNA damage, and MK-8776 is known to abrogate cell cycle arrest and aberrant mitosis. Here, we found that the chemosensitizing ability of MK-8776 is associated with the inhibition of gemcitabine-induced CHK activation and the promotion of gemcitabine-induced DNA damage. In particular, MK-8776 increased CHK1 phosphorylation at Ser345 (ATR-mediated CHK1 phosphorylation) and enhanced DSBs induced by gemcitabine. This is likely associated with its inhibitory effects on homologous recombination, a DSB repair mechanism that uses the undamaged sister chromatid as a repair template during intra-S or G2 phase^20^.

DNA is constantly exposed to a wide range of genotoxic agents. To overcome DNA damage, cells have evolved a complex mechanism known as the DNA damage response, which detects damage and promotes DNA repair^21^. DNA single-strand breaks are discontinuities in one strand of the DNA duplex that are detected by ATR, which subsequently activates the downstream protein CHK1. Unrepaired single-strand breaks can lead to DNA replication stress and be converted into DSBs during S-phase, resulting in genome instability^22^. DNA damage responses, particularly ATR-CHK1 signaling, play an important role in therapeutic responses following chemotherapy^23,24^. Selective CHK1 inhibitors may selectively sensitize p53 defective cancer cells to the cytotoxic effects of gemcitabine since p53 also plays a major role in checkpoint function for many anticancer therapeutics that cause DNA damage^10,25,26^. Tumor cells with mutated p53 display G1 checkpoint defects and thus rely on the S or G2 checkpoint involving CHKs for DNA repair^10^. Consequently, CHK inhibition can force p53-defective tumor cells to enter mitosis with unrepaired DNA damage, whereas p53-wild type containing non-tumor cells can overcome this stress by promoting the G1 checkpoint^10,25^. Notably, p53 function is lost in almost all invasive bladder cancer cells^12^, thus contributing toward the unusual mode of cell death caused by defective cell cycle checkpoint regulation observed in this study. Conversely, Montano et al*.* reported that the gemcitabine-sensitizing effects of CHK1 inhibitors were independent of p53 status^27^. Therefore, future studies should identify novel biomarkers to detect DNA repair defects in bladder cancer cells and thereby predict the response to CHK1 inhibitors.

A phase-I clinical trial showed that MK-8776 is well tolerated with good clinical efficacy both as a monotherapy and in combination^17^; however, the results of a phase II trial have not yet been announced. Previous studies have shown that MK-8776 can sensitize cells to radiotherapy or histone deacetylase inhibitors, which induce DNA DSBs, via the CHK-related DNA damage response and cell cycle regulation^28,29^. Moreover, MK-8776 has been shown to restore sensitivity to chemotherapeutic agents in P-glycoprotein-overexpressing cancer cells or re-sensitize cancer cells to the effects of different therapies by inhibiting autophagy^30,31^. However, the exact mechanism by which MK-8776 affects the response to gemcitabine in bladder cancer remains unknown. The in vitro experiments performed in this study revealed that the combination treatment increased the expression of histone H2A.X phosphorylated at Ser 139, yet MK-8776 alone had little effect on γH2A.X. In addition, the co-administration of the four bladder tumor cell lines with MK-8776 and gemcitabine resulted in persistent DNA damage for at least 48 h. To our knowledge, there are currently no clinical trials of MK-8776 in bladder cancer patients, despite these encouraging preclinical results which suggest that its combined use with gemcitabine may be more effective and better tolerated in patients with advanced bladder cancer.

Several previous reports have indicated that p21^CIP1^ regulates transcription in DNA damage response mechanisms, including homologous recombination and cell cycle arrest^32^. Since p21^CIP1^ is a potent cyclin-dependent kinase (CDK) inhibitor that promotes homologous recombination, increased CDK activity and the inhibition of cell cycle arrest in the absence of p21^CIP1^ increase DNA damage^33^. In this study, the observed changes in p21^CIP1^ expression were cell line dependent; indeed, 5637 was the only cell line examined in which p21^CIP1^ expression increased following the combination treatment, despite elevated levels of DNA damage. This finding may indicate that MK-8776 suppresses the effect of p21^CIP1^ in the DNA repair process in 5637 cells; however, further studies are required to elucidate the role of p21^CIP1^ in bladder cancer cells in response to CHK1 inhibition.

Despite the important findings, our study had limitations. Firstly, we investigated the effects of pharmacological CHK1 inhibition in four bladder cancer cell lines representing the heterogeneity of bladder cancer; thus, our findings cannot simply be extended to all bladder cancer cells. Further investigations are also required to clarify whether the cytotoxic effect of the combination treatment is related to p53 function or any other characteristics of the cells, including non-tumor cells.

In conclusion, we found that the combination of gemcitabine and MK-8776 exerted strong antitumor effects on bladder cancer cells. Thus, our study demonstrates the promise of agents that target checkpoint kinases and the DNA replication stress response as a therapeutic strategy to treat bladder cancer. As such, we support the development of this approach to investigate CHK1 inhibitors combined with gemcitabine in clinical studies for patients with advanced bladder cancer.

## Methods

### Cell culture and reagents

Four human bladder cancer cell lines (5637, J82, T24, and UM-UC-3) were obtained from the American Type Culture Collection (Rockville, MD, USA) and cultured in RPMI-1640, MEM, or McCoy's 5A medium containing 10% FBS and 1% penicillin/streptomycin (Invitrogen, Carlsbad, CA, USA) at 37 °C in a 5% CO_2_ atmosphere. All cell lines used in the study were p53 mutated. Gemcitabine and MK-8776 were purchased from Selleck Chemicals (Houston, TX, USA).

### MTS assay

Cancer cells were seeded onto 96-well plates at a density of 3 × 10^3^ cells/well, allowed to attach for 24 h, and then treated with different concentrations of gemcitabine and/or MK-8776. Cell viability was measured after 48 h using an MTS assay by measuring color intensity with a plate reader at 490 nm (CellTiter 96 Aqueous kit; Promega, Madison, WI, USA) as previously described^34^.

### Colony formation assay

Cells were seeded onto 6-well plates at a density of 3 × 10^2^ cells/well, allowed to attach for 24 h, and then treated with 10 nM gemcitabine and/or 0.5 μM MK-8776 for 24 or 48 h. The cells were then supplied with fresh media and allowed to grow. After 10 days, formed colonies were fixed in 100% methanol and stained with 5% Giemsa (Muto, Tokyo, Japan) for 15 min. Absorbance was measured at 560 nm.

### Flow cytometry

For flow cytometry, 2.5 × 10^4^ cells were cultured in a 6-well plate for 24 h and then treated with different concentrations of gemcitabine and/or MK-8776 for 24 or 48 h. To analyze the cell cycle, detached cells in the supernatant and adherent cells were harvested and stained with propidium iodide. For the annexin V assay, harvested cells were stained with annexin V-FITC to detect apoptosis and with 7-AAD to detect necrosis following the manufacturer’s instructions (Beckman Coulter, Marseille, France). Flow cytometry data were acquired and analyzed using CellQuest Pro software (BD Biosciences, San Jose, CA, USA).

### In vivo experiments

All procedures carried out in this study were approved by the Institutional Animal Care and Use Committee of the National Defense Medical College (approval number 14068). The study was carried out in compliance with the ARRIVE guidelines. Subcutaneous tumor nodules were generated by injecting UM-UC-3 cells (1 × 10^7^ cells) into the flanks of five-week-old male BALB/c Slc-nu/nu mice obtained from CLEA (Tokyo, Japan) 5 days before treatment initiation. Mice were then intraperitoneally administered gemcitabine (20 mg/kg) and/or MK-8776 (4 mg/kg) or the vehicle control (DMSO) once daily from day 1 (*n* = 5 per group) for two weeks. Mice were carefully monitored, and two perpendicular diameters of the subcutaneous tumor were measured with calipers every two or three days. Tumor size was determined by calculating tumor length and width (tumor volume = 0.5 × length × width^2^), and differences between the four treatment groups were analyzed using the Mann–Whitney U test. Body weight was measured every week. Mice were killed on day 15 according to ethical policies for animal research^35^. All applicable international, national, and institutional guidelines for the care and use of animals were followed.

### Western blot analysis

Total protein lysates were obtained using RIPA-buffer (0.1% sodium dodecyl sulfate (SDS), 0.5% deoxycholate, 1% Nonidet P-40, 150 mM NaCl, 1% Triton X-100, 50 mM Tris (pH 7.6), 1 mM EDTA, and 10 μL/mL protease inhibitor cocktail; Sigma Aldrich, St. Louis, MO, USA). Equal amounts of protein from each sample were loaded onto SDS-PAGE gels and wet-blotted onto nitrocellulose membranes which were then blocked with 5% BSA or skimmed milk in TBS with 0.2% Tween-20 (TBS-T) and incubated with the following primary antibodies at 4 °C overnight: anti-CDK 4, anti-cyclin D1, p21^CIP1^ (1:250, Santa Cruz Biotechnology, Santa Cruz, CA, USA), active caspase 3 (1:500, Epitomics, Burlingame, CA, USA), PARP, cleaved PARP, phosphorylated CHK1 (Ser345), CHK1, pH2A.X, and Rad51 (1:1000, Cell Signaling Technology, Danvers, MA, USA). Anti-actin antibodies (1:3000 dilution; Millipore, Billerica, MA, USA) were used as a loading control. Membranes were washed three times in TBS-T and incubated with horseradish peroxidase-labeled secondary antibodies (1:6000 dilution; Bio-Rad, Hercules, CA, USA) for 1 h before being washed again. Labeled proteins were detected using an enhanced chemiluminescence detection system (ECL Plus Western Blotting Detection System; GE Healthcare, Wauwatosa, WI, USA). Protein amounts were calculated relative to the actin reference using the ImageJ plugin, ColonyArea, developed by Guzmán et al.^36^.

### Statistical analysis

The Chou–Talalay method was used to calculate combination indices (CalcuSyn software, Biosoft, Cambridge, UK). The Mann–Whitney U test was used to analyze differences between groups in StatView software (SAS Institute, Cary, NC, USA). Differences were considered significant for *p* values of < 0.05.

## Supplementary Information

Supplementary Information.

## Data Availability

The datasets generated during and/or analyzed during the current study are available from the corresponding author on reasonable request.
