# Supplementary material for: Inhibition of checkpoint kinase 1 potentiates anticancer activity of gemcitabine in bladder cancer cells
Source: Sci Rep. 2021 May 13;11:10181. doi: 10.1038/s41598-021-89684-5 (PMC8119486; doi:10.1038/s41598-021-89684-5)
Supplement: Supplementary file 1 — Supplementary Information. [file 41598_2021_89684_MOESM1_ESM.docx]

**Inhibition of checkpoint kinase 1 potentiates anticancer activity of gemcitabine in bladder cancer cells**

Makoto Isono*, Kazuki Okubo, Takako Asano & Akinori Sato

Department of Urology, National Defense Medical College, Namiki, Tokorozawa, Japan

***Corresponding author:**

Makoto Isono, Department of Urology, National Defense Medical College, 3-2 Namiki, Tokorozawa, Saitama 359-8513, Japan

Phone: +81-4-2995-1676; Fax: +81-4-2996-5210; E-mail: mktisn@hotmail.com


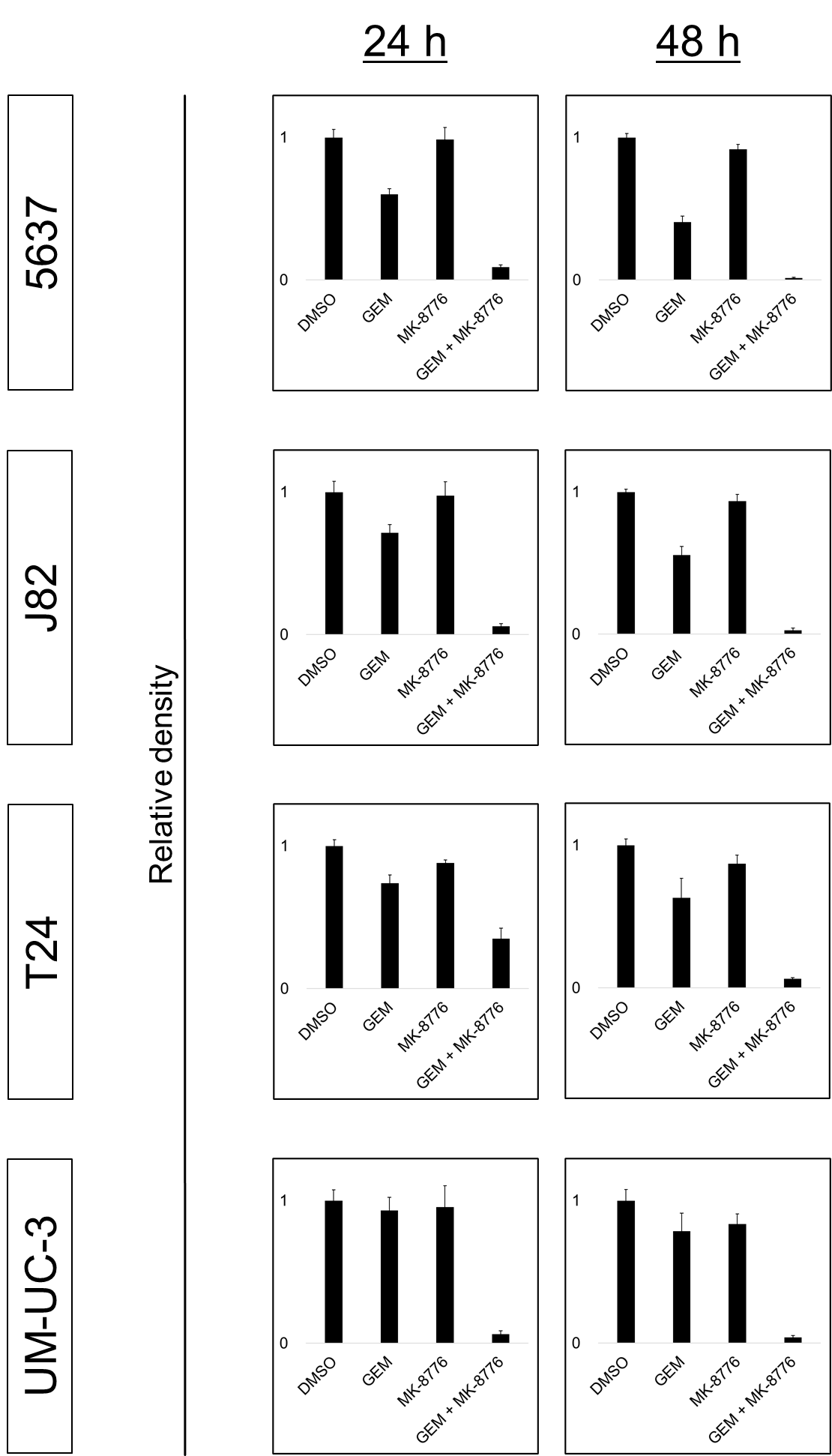


**Supplementary Fig. S1** Colony formation assay following gemcitabine (10 nM) and/or MK-8776 (0.5 μM) treatment for 24 or 48 h. DMSO was used as a negative control. Bar graphs show the relative density of the cells at each treatment module.


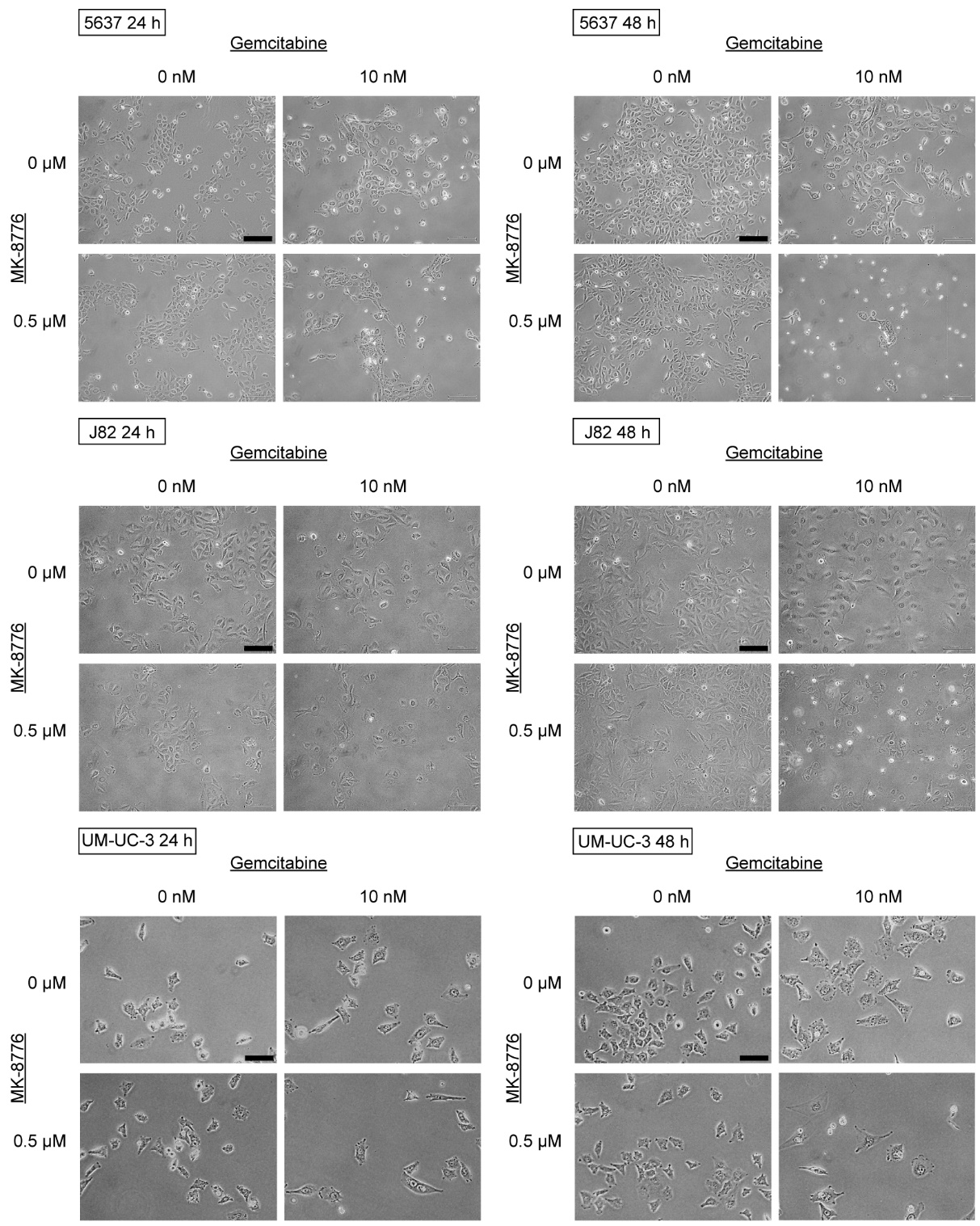


**Supplementary Fig. S2** Morphology of the 5637, J82, and UM-UC-3 cells visualized by light microscopy with or without gemcitabine and MK-8776 treatment at the indicated concentrations. Scale bar: 100 μm.


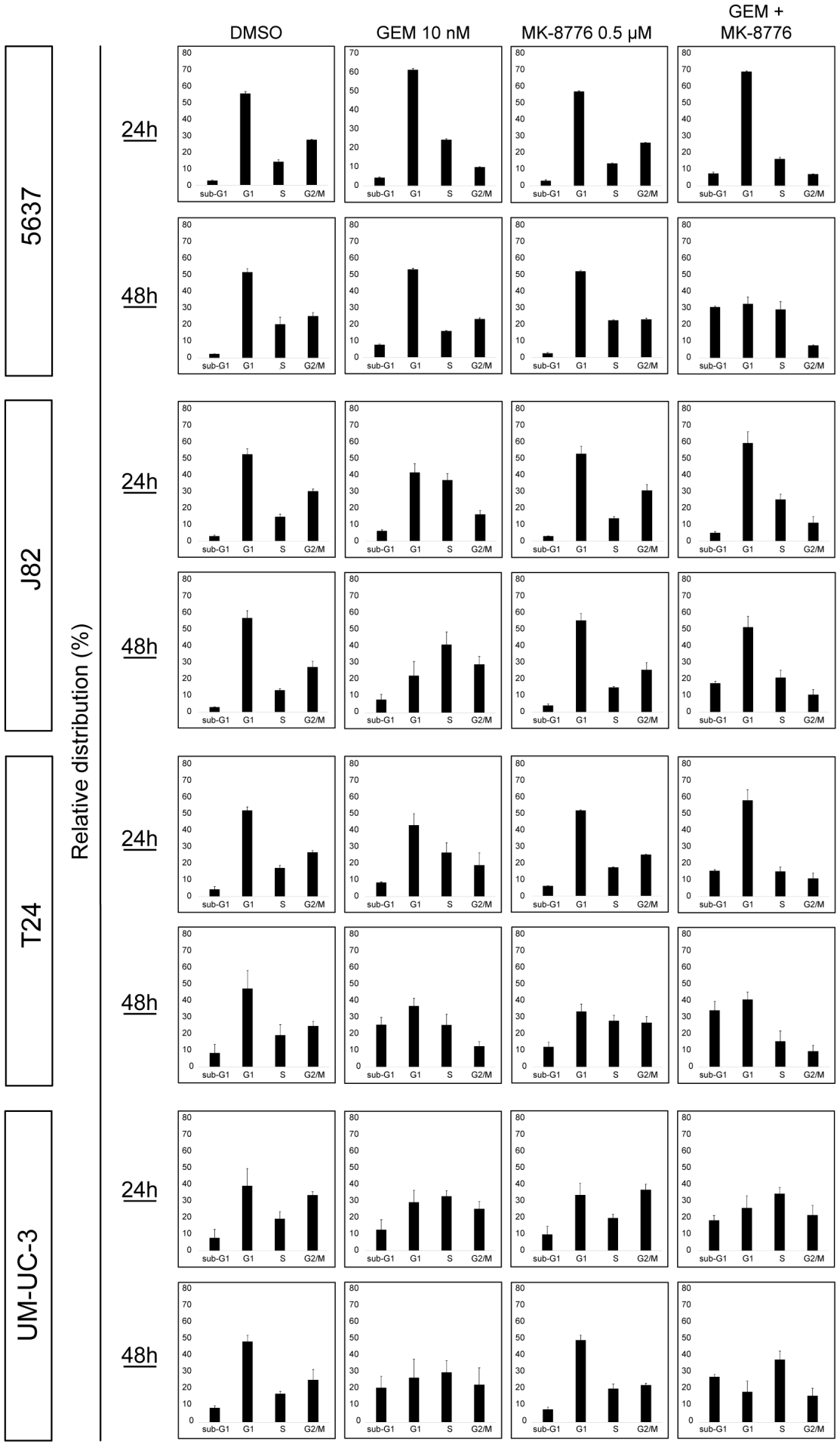


**Supplementary Fig. S3** Cell cycle analysis by flow cytometry following gemcitabine (10 nM) and/or MK-8776 (0.5 μM) treatment for 24 or 48 h. DMSO was used as a negative control. Bar graphs show the relative distribution of the cells at each phase of the cell cycle.


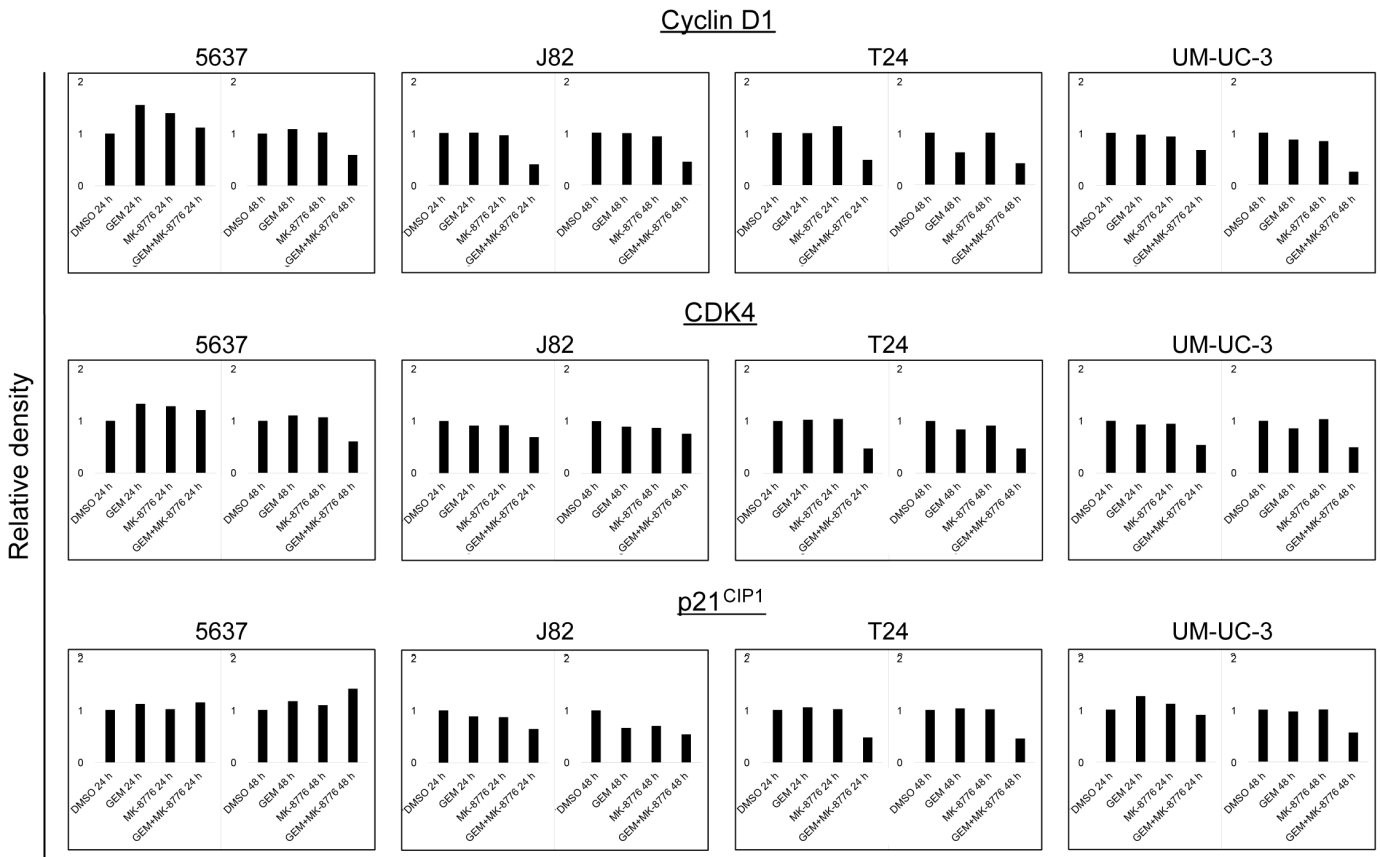


**Supplementary Fig. S4** Expression of cell cycle-associated proteins evaluated by western blotting following gemcitabine (10 nM) and/or MK-8776 (0.5 μM) treatment for 24 or 48 h. Actin was used as a loading control. The relative densitometry quantification of the signals is illustrated.


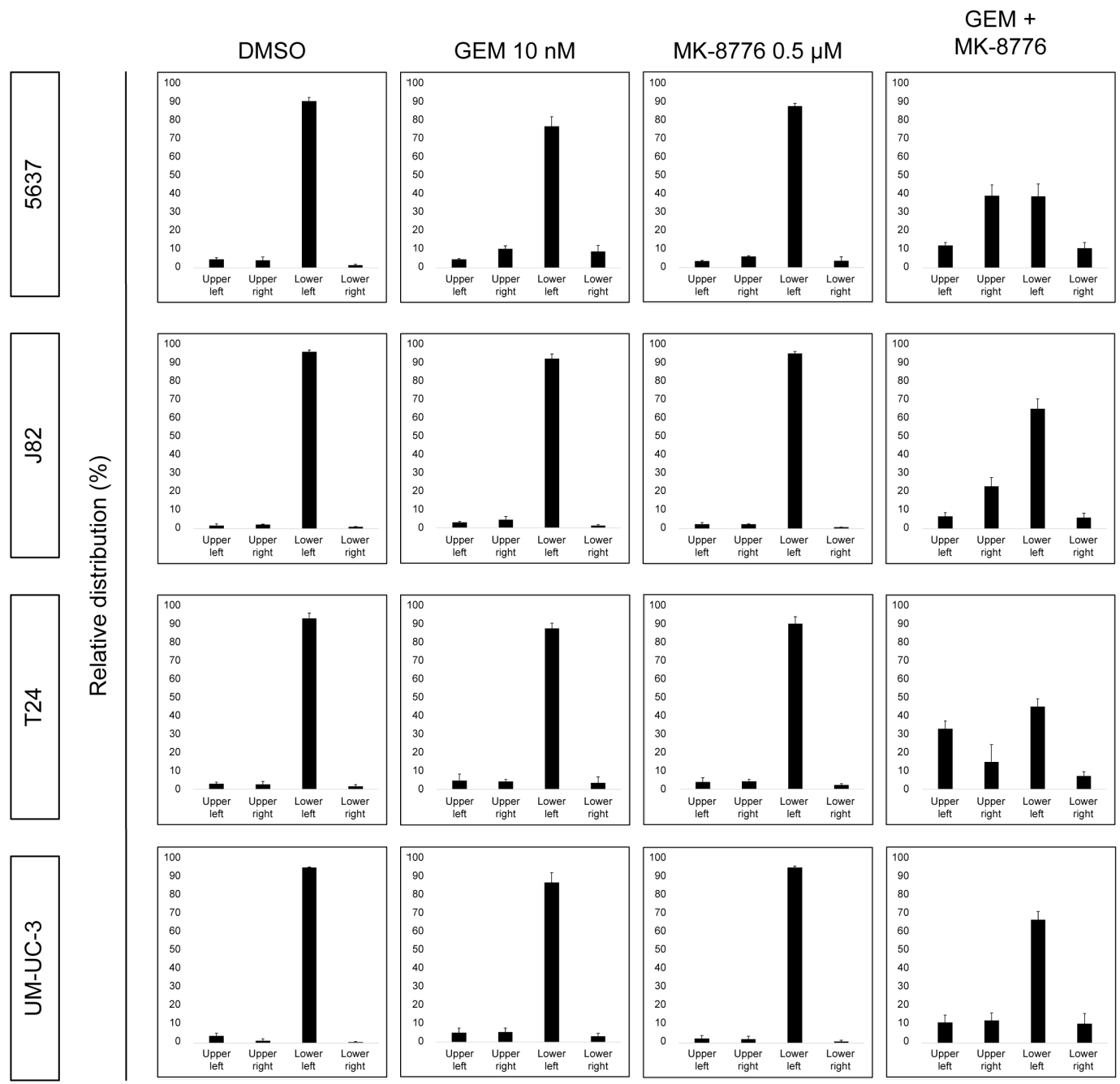


**Supplementary Fig. S5** Flow cytometric analysis of bladder cancer cells treated with indicated conditions after combined staining with Annexin V and 7-AAD. The results are expressed as a percentage of early apoptotic cells (lower right quadrant), late apoptotic cells (upper right quadrant), and necrotic cells (upper left quadrant). Bar graphs show the relative distribution of the cells at each quadrant.


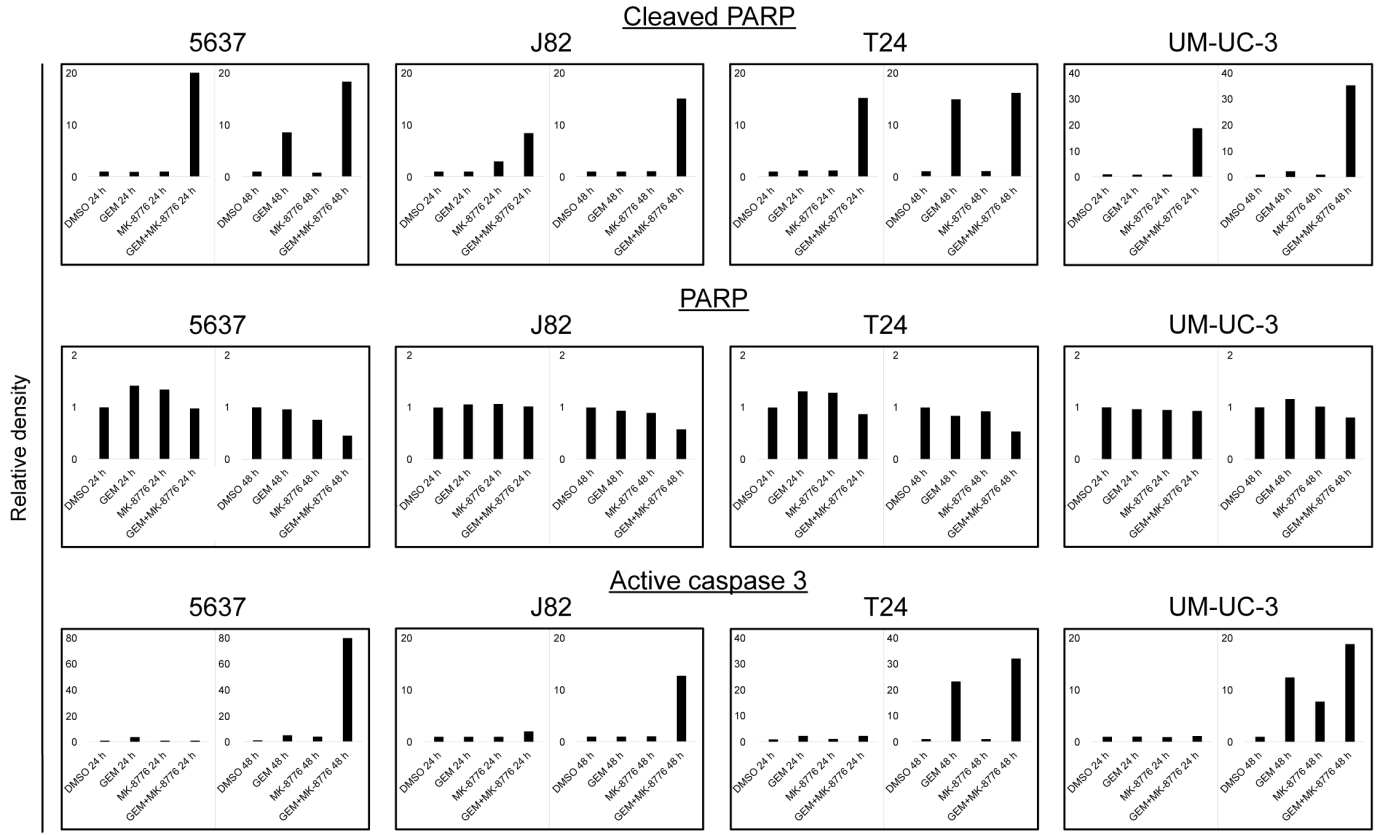


**Supplementary Fig. S6** PARP cleavage and active caspase 3 evaluated by western blotting following gemcitabine (10 nM) and/or MK-8776 (0.5 μM) treatment for 24 or 48 h. Actin was used as a loading control. The relative densitometry quantification of the signals is illustrated.


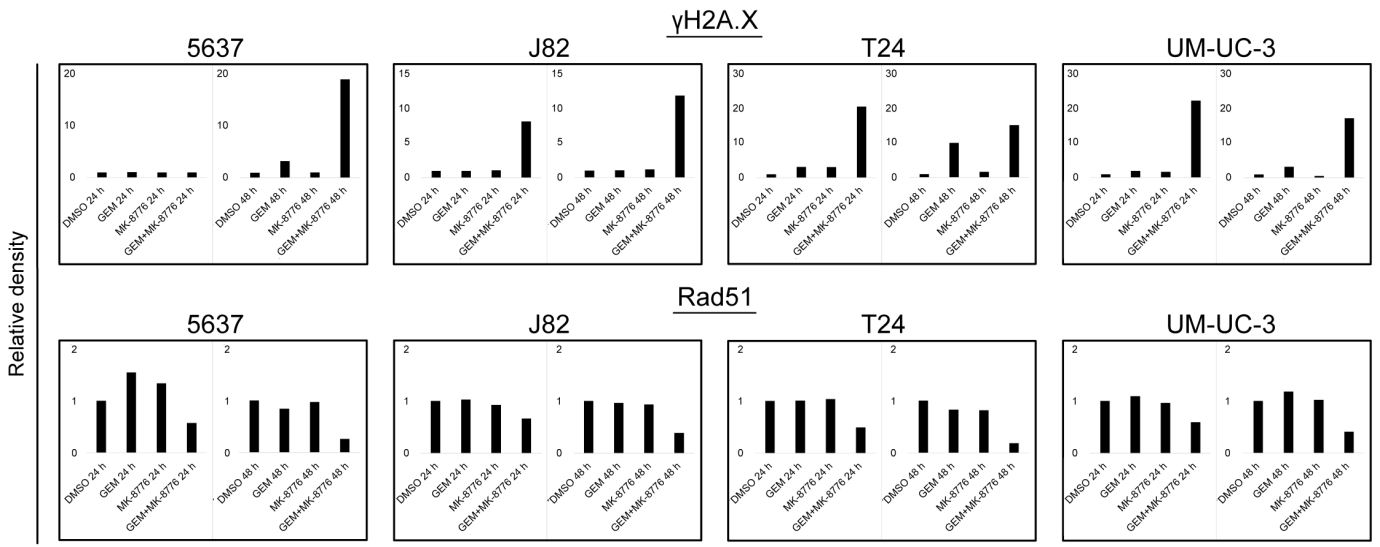


**Supplementary Fig. S7** γH2A.X and Rad51 levels evaluated by western blotting following gemcitabine (10 nM) and/or MK-8776 (0.5 μM) treatment for 24 or 48 h. Actin was used as a loading control. The relative densitometry quantification of the signals is illustrated.


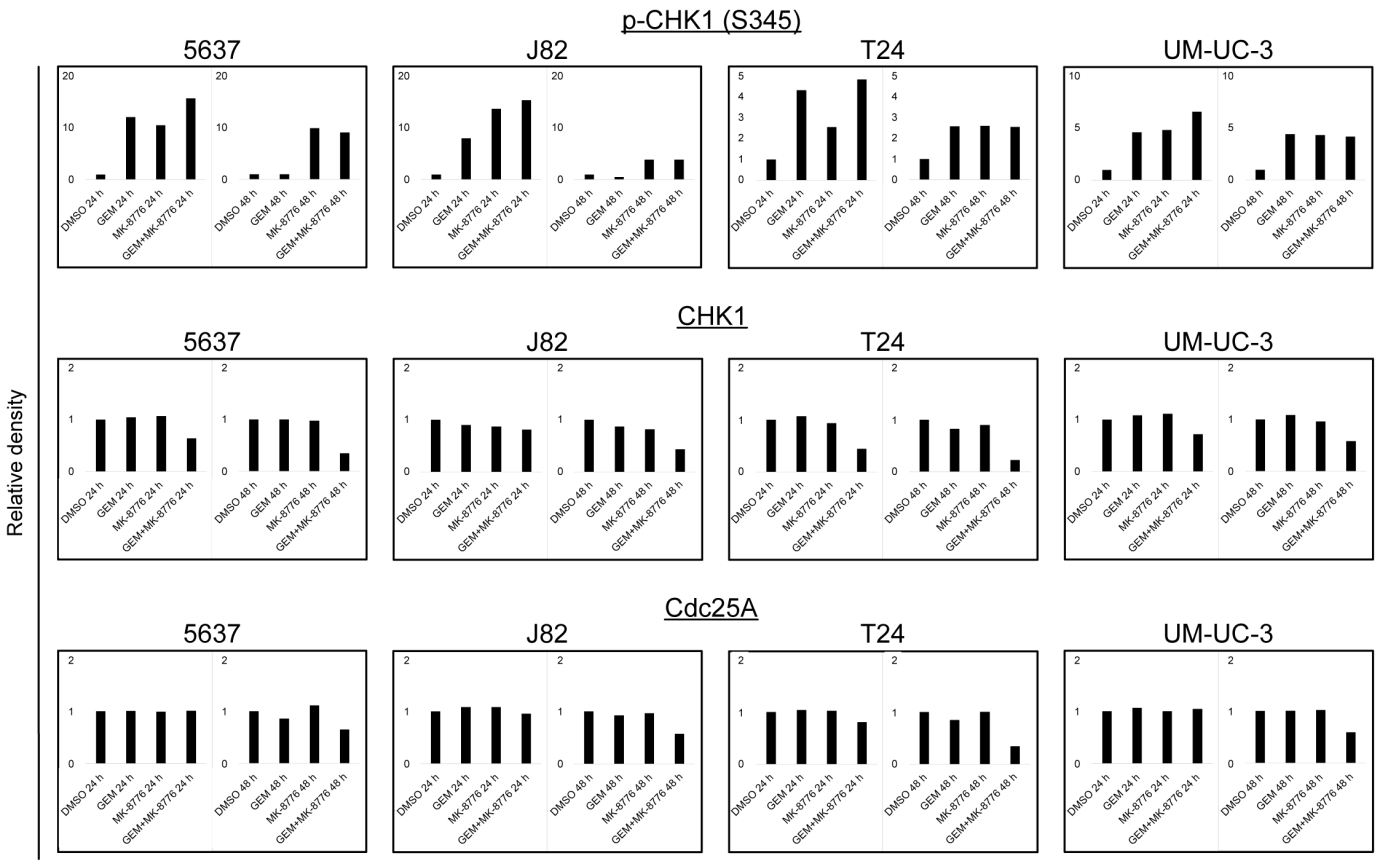


**Supplementary Fig. S8** Expression of the indicated cell cycle checkpoint-related proteins evaluated by western blotting following gemcitabine (10 nM) and/or MK-8776 (0.5 μM) treatment for 24 or 48 h. Actin was used as a loading control. The relative densitometry quantification of the signals is illustrated.


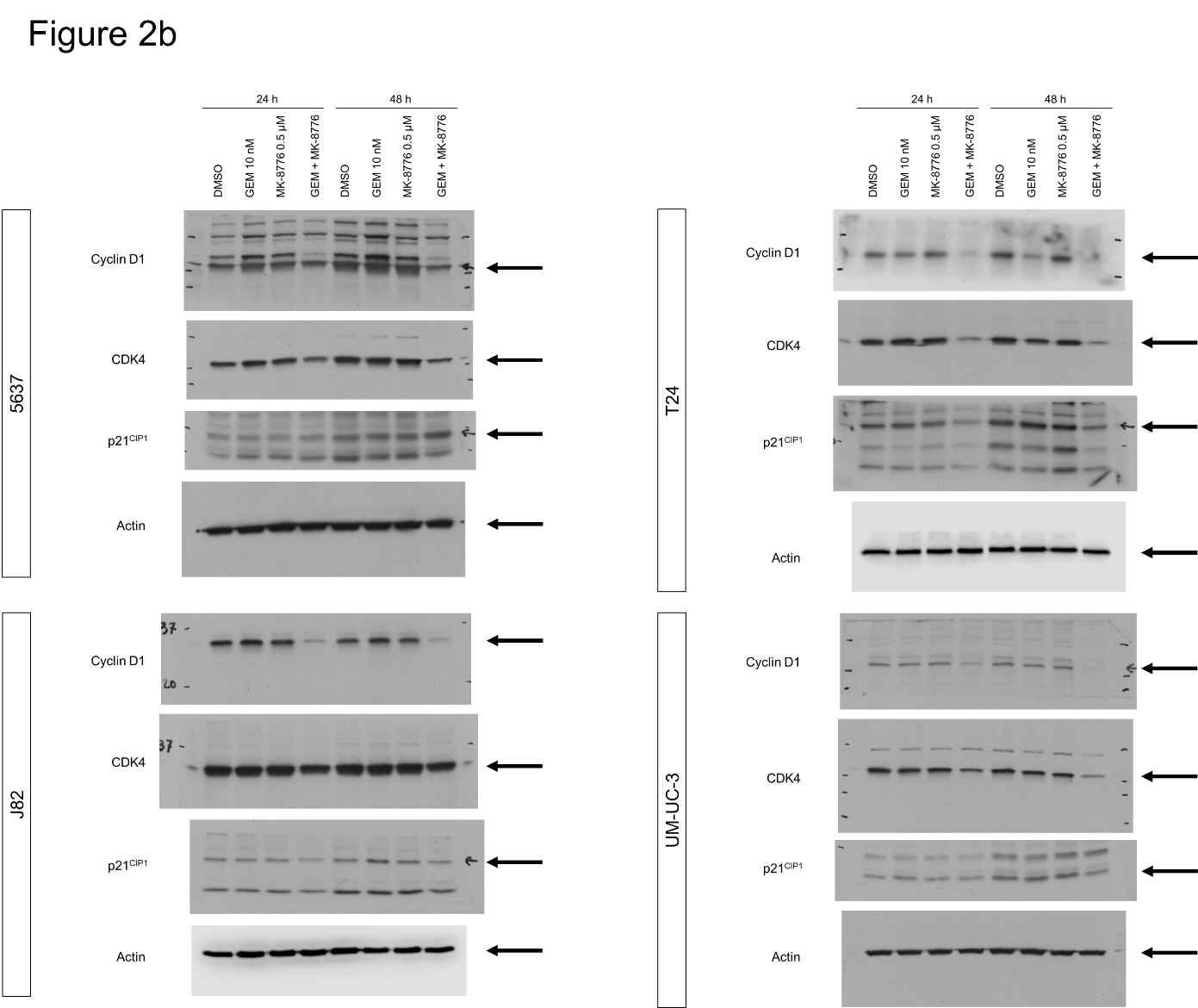


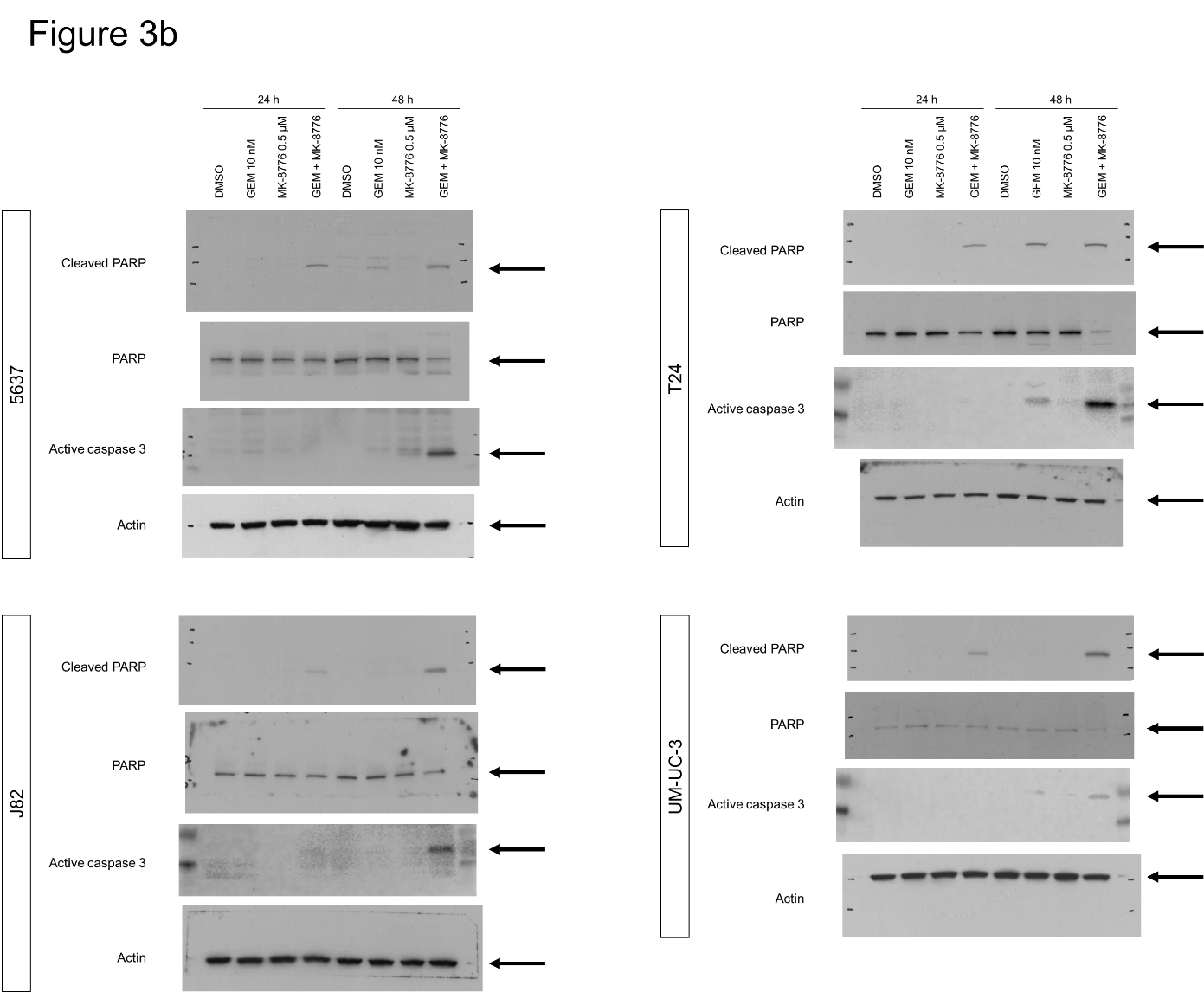


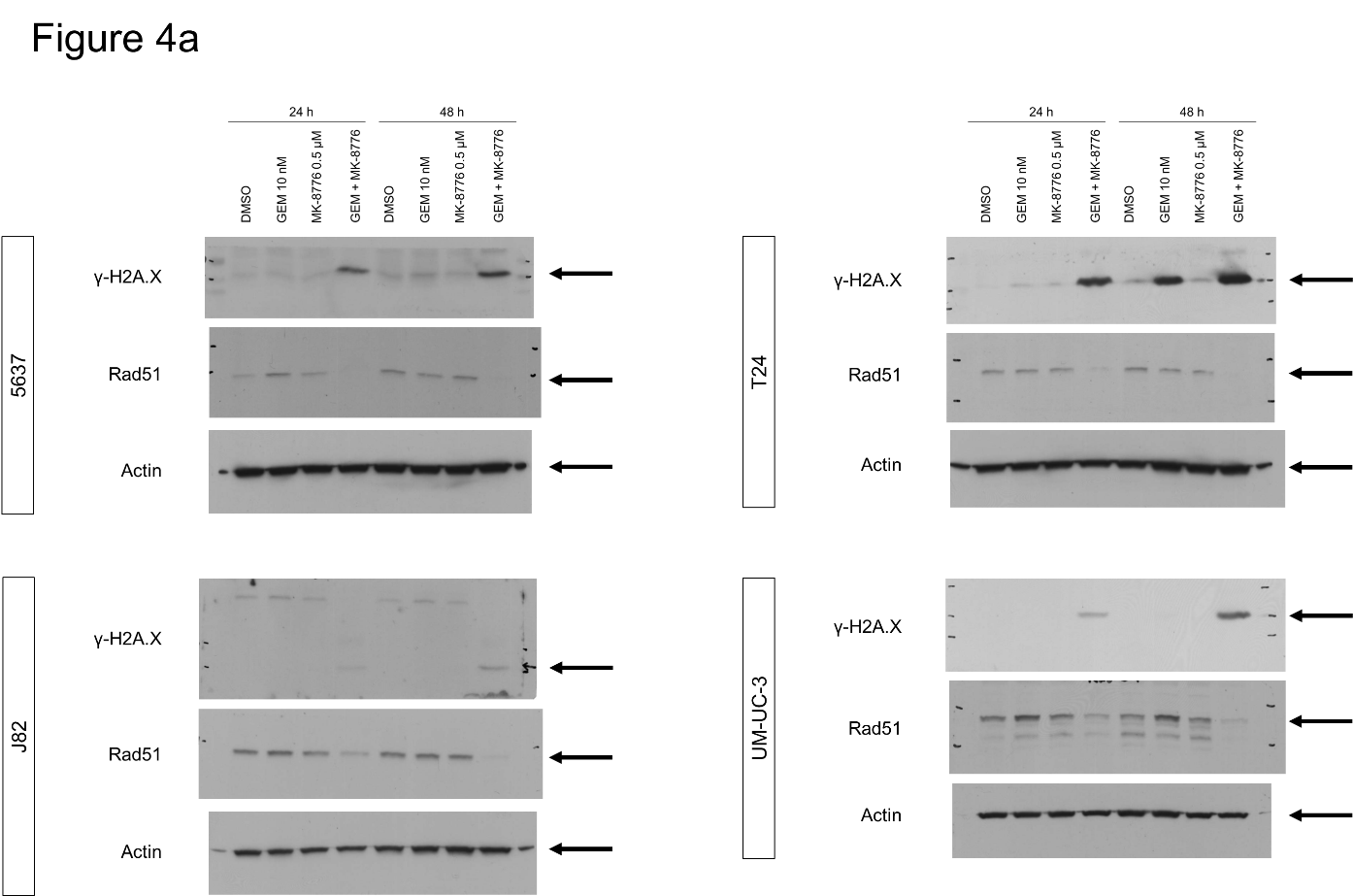


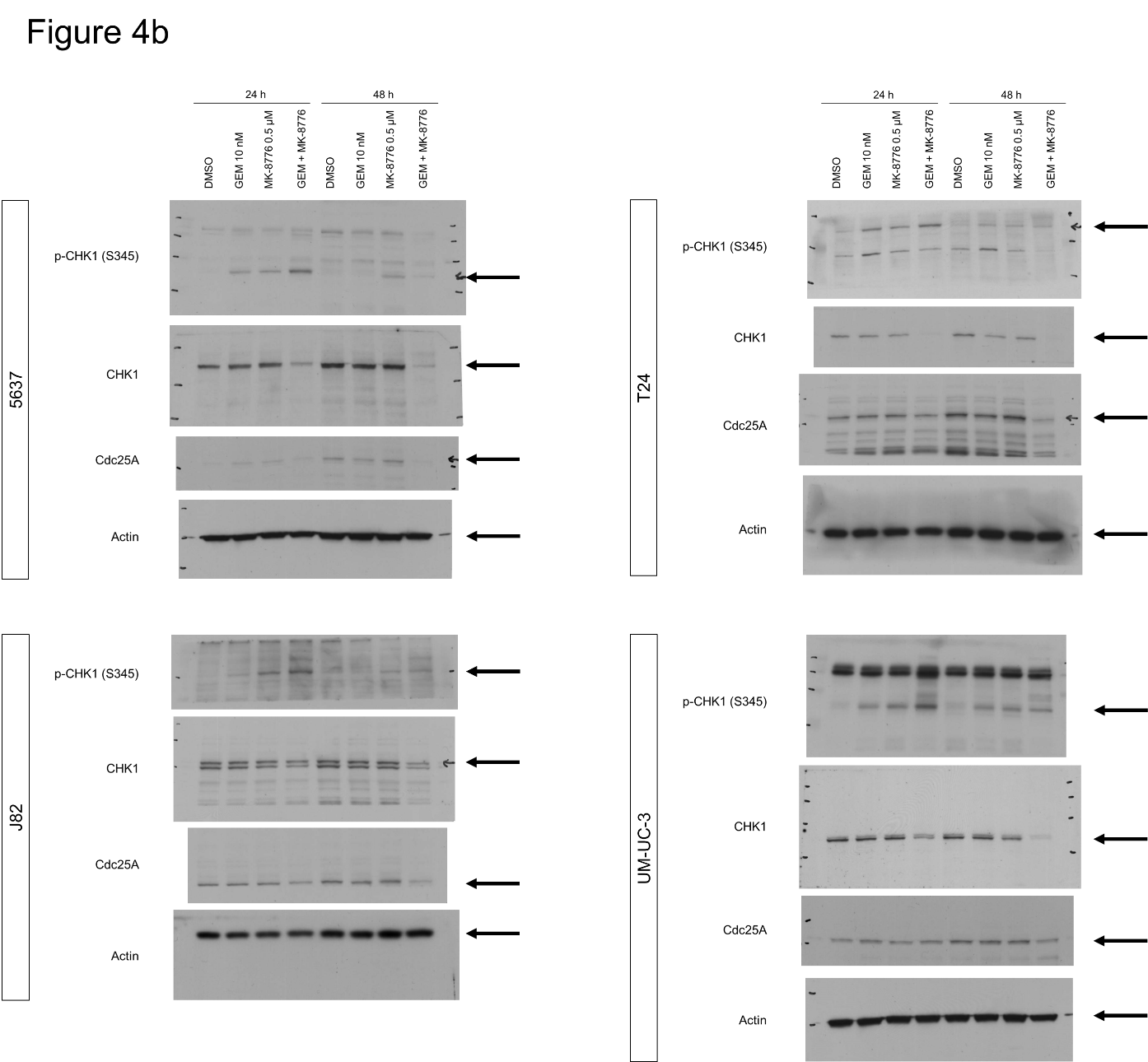


**Supplementary Fig. S9** Uncropped blots corresponding to Figure 2b, 3b, 4a, and 4b. Arrows indicate cropped bands. Note that the membranes were cut before probing.
